# Systematic review and meta-analysis assessing the knowledge and use of the female condom among Nigerians

**DOI:** 10.4314/ahs.v21i3.48

**Published:** 2021-09

**Authors:** Philemon Dauda Shallie, Firoza Haffejee

**Affiliations:** 1 Department of Basic Medical Sciences, Durban University of Technology, KwaZulu-Natal, South Africa; 2 Department of Anatomy, Olabisi Onabanjo University, Ogun State, Nigeria

**Keywords:** Female condom, women, men, awareness, use, Nigeria

## Abstract

**Background:**

The female condom (FC) is a critical component in a comprehensive and sustainable approach to prevent HIV, other sexually transmitted infections and unintended pregnancies.

**Objectives:**

This review provides comprehensive information about Nigerian's knowledge and use of FC.

**Methods:**

We screened search output, evaluated study eligibility, and extracted data in duplicate. Data from similar studies were combined in a meta-analysis.

**Results:**

There was a significantly (p < 0.0001) high-level of awareness amongst the respondents. However, the use of the FC was very low at 5.5% among female respondents. There was a significant (p < 0.0001) difference between FC awareness and use. The main reasons for FC use were prevention of unintended pregnancy (55%) and STIs/HIV (31%). We observed a significant difference between reasons of non-use of the FC [F (5, 13) = 5.195, P = 0.0077]. Furthermore, there were significant differences between the sources of information on FC [F (3, 8) = 32.89, P < 0.0001].

**Conclusion:**

Despite the high levels of awareness, especially among the female respondents, the use of the FC has remained extremely low even among the young, educated undergraduate students. There is aneed for robust and consistent advocacy to make the FC available and affordable.

## Introduction

Global surveys by United Nations in 2019 showed that globally of the 1.1 billion women who want to avoid pregnancy, 10% do not use contraceptives 1. Over the last two decades there has been a change in the type of contraceptive methods used. For instance, the global prevalence of female sterilisation has declined from 13.7 per cent in 1994 to 11.5 per cent in 2019. Although, the use of the pill, IUD and rhythm methods have remained relatively stable over the past 25 years, the use of male condoms have more than doubled worldwide from 4.5% in 1994 to 10.0% in 2019. In sub-Saharan African countries, the prevalence of implants (Levonorgestrel and Etonogestrel), injectable contraceptives and male condoms has increased 1.

The pill and the barrier contraceptives have their own unique advantages and disadvantages; condoms offer far more protection against STDs than hormonal forms of birth control, which offer no protection against sexually transmitted disease (STD) at all, however the pill is slightly more effective at preventing pregnancy than condoms. They also have several complementary benefits; using condoms and the pill simultaneously further lowers the risk of pregnancy while offering protection against most STDs, making them a good combination when used together. Nevertheless, condoms have a range of advantages over other forms of birth control, from their low cost to their ability to prevent the spread of most STDs 2.

Despite the reported gradual decline in the HIV epidemic, it has remained a cause of great concern for developing countries in sub-Saharan Africa, where twothirds of all infected persons live, and three-quarters of global deaths occur 3,4. The current Nigerian national HIV prevalence among adults aged 15–49 years is 1.9 million people (1.4%), this is more than twice in females (1.9%) than in males (0.9%). Younger women aged 20–24 are more than three times likely to be living with HIV than younger men of the same age group. The prevalence varies across the geopolitical regions; South-South (3.1%), North-Central (2.0%), South-East (1.9%), South-West (1.1), North-East (1.1%), and North-West (0.6%) 5. It was also stated that the prevalence of STDs among young Nigerian females is 17% and that these increase the risk of both female infertility as well as transmission of HIV/AIDS 6. Furthermore, adolescent pregnancy is a daunting problem in Nigeria. Studies have shown the prevalence of unintended pregnancy among adolescents range between 23% and 36% in the different regions of the country 6.

The global effort to curb the spread of HIV and other sexually transmitted infections (STIs), has resulted in the introduction of condoms 7. These are a significant component in a comprehensive and sustainable approach to prevent HIV and other sexually transmitted infections (STIs) and are effective in preventing unintended pregnancies 8. The female condom (FC) was introduced as a female-initiated protective barrier that prevented pregnancy and HIV as well as other STIs and its uptake was regarded as safe 7,9. Although, the FC sought to provide women with the ability to have a controlled decision of sexual protection, its use is not entirely female-controlled, as a woman needs the co-operation of her male partner 9. Nigerian Demographic and Health Surveys (DHS) 2008, statistics showed that although 14.7% of all women had heard about the FC and 13.9% had heard a specific family planning message on the FC, only 0.2% had ever used one 10,11. The promotion of the FC remains important, despite both successes and disappointments, especially in the face of heterosexually acquired HIV infection rates that are soaring globally 9.

In the baseline study conducted in 2011 by the Society for Family Health (SFH), Nigeria, 38.9% of respondents had heard about FCs, but only 3.5% of these had ever used one. Thus 1.4% of all respondents had ever used an FC and 1.7% of the male respondents reported that their female sex partners used FC compared to 1.0% among the female respondents 11. Most youths in Nigeria and elsewhere are sexually active and engage in risky sexual behaviours, such as early sexual debut, unprotected sex, multiple sexual partnerships and anal sex. In addition, they have incorrect knowledge of STIs 12–14. Furthermore, they lack the confidence to negotiate sex or contraceptive use. At the same time, many do not perceive themselves as being at risk of contracting STIs, including HIV and AIDS 14–16.

Correct and consistent use of condoms, including the FC, is one of the best ways to prevent STIs, including HIV and AIDS 17. Some national and international agencies like Universal Access to FCs (UAFC), have set out objectives to increase public awareness to create FC demand and ensure availability of FCs in order to reduce the number of unintended pregnancies and subsequently reduce maternal deaths, as well as to reduce the preponderance of HIV and sexually transmitted infections (STIs) 11.

This review provides comprehensive and current information about the knowledge and use of FC among Nigerians of reproductive age, in order to stimulate research interest geared to address the existing FC programs and to enhance its acceptance and use in the country.

## Methodology

**Fig. 1:**
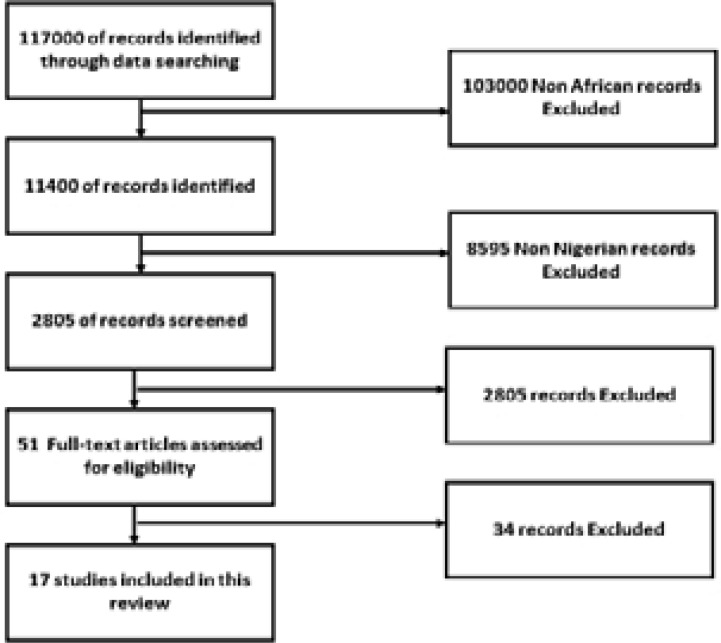
Flow Diagram of Literature Selection

The preferred reporting items for systematic reviews and meta-analysis (PRISMA) model was used to develop the review process 18. We searched the literature in two databases: Google Scholar and PubMed for literature and earlier reviews. Screening titles and abstracts for combinations of the terms “female condom,” “use among women in Nigeria,” and “male perception.” In order not to miss out on publications, we included words in our search terms that might indicate acceptability or its opposite, such as accept(ance), adopt(ion), attitude (attitudinal). A total of 117,000 search hits were generated, which was scaled down to 11,400 when the search was limited to Africa and subsequently scaled down to 2,805 when the search was limited to Nigeria. From these, 51 published articles and reviews were carefully examined and filtered by the authors. Of these, only 17 studies met the inclusion criteria for the study (see Fig. 1). The search mode used was the Boolean search. The following search terms were used to filter the required studies: “condom use,” “Nigeria,” “woman or female or girls,” and “female condom”.

### Inclusion and exclusion criteria

We searched literature published between 2000 and 2020, with the following eligibility criteria: (1) published in the English language and comprised of either quantitative or qualitative research design; (2) retrievable, as a full study, a review, or as an article in peer-reviewed scientific journals; (3) have a sound scientific methodology; (4) study female knowledge, acceptability, attitude or perception of use of the FC; (5) report on women in Nigeria and (6) studies with both male and female perspectives where each perspective was reported separately and could be extracted from the studies. We found 51 studies, 17 of which met and 34 did not meet all pre-set criteria.

The exclusion criteria ranged from dual country comparative studies, which were excluded due to cross-tabulation of results and mixed-gender studies, where the female perspective could not be extracted. Seventeen eligible studies were subjected to an in-depth analysis.

The following information was extracted from the articles: author names, year of publication, study population, study design, sample size, outcome measures, and results. Studies are presented in chronological order in Table 1. Outcome measures that were common in the studies were identified and used to create themes to understand Nigerian women's current awareness and FC use.

### Patient and Public Involvement (PPI) statements

This systemic review was done without patient involvement. Patients were not invited to comment on the study design and were not consulted in the development of relevant outcomes or the interpretation of the results. Patients were not invited to contribute to the writing or editing of this document for readability or accuracy.

## Results

Figure 1 summarises the search and selection process. We generated 117,000 search hits, which were scaled down to 11,400 when the search was limited to Africa and subsequently scaled down to 2,805 when the search was limited to Nigeria. The authors carefully examined and filtered 51 published articles and reviews. Of these, only 17 studies met the inclusion criteria for the study. The authors analyzed one retrospective review, two retrospective surveys, thirteen cross-sectional studies, and one qualitative study. Common themes from the outcome assessments of each study were derived and are explained in detail below. These themes are summarized and compared in the chronological lists in Tables 1a and 1b.

**Table 1a:** Summary of Studies Investigating Female Condom (FC) Use among Nigerian Women

Study No.	Authors/ Year	Study Design	sample size	Study Duration	Outcome Assessment	Results
1	Uchendu, O. C., Adeyera, O., & Owoaje, E. T. (2019)	Cross sectional study	964 674-M 290-F	Not specified	Awareness and utilization of FC predictors of FC awareness and utilization.	Almost half 47.9% of the respondents have heard about the FC however only 16.8% have ever seen one while 4.3% have ever used an FC. Age, education, current sexual activity and experience of rape attempt were predictors of FC awareness.
2	Adinma, J., Adinma, E., Eke, N., & Umeononihu, O. (2016).	Cross sectional study	276 168-M 108-M	Not specified	Awareness and use FC; reasons for nonuse of FC; indication for FC use; awareness of functions of FC knowledge of the pitfalls of FC; and source of information of FC.	About 62.3% were aware of FC. 5.1% respondents have ever used the FC. Reason for non–use of FC were religious belief 3.3%, not knowing how to use FC 20.5% and preference for other methods of contraception. 7.7%. Lack of money was the reason for nonuse by 5.1% respondents. Many of the respondents 50.5% used FC when afraid of STI or pregnancy. However, few others used it only during sex with a non-regular partner 22.1%) and only when requested by partner 12.6%.
3	Usman, S. O., Kalejaye, O. O., Isola, I. N., Oluwaniyi, O., Ojogbede, A. K., & Adu, A. S. (2016)	Cross sectional study	1500		FC use, reasons for use and source of information.	FC use is 2.9% some of the respondents believe that contraception prevents unwanted pregnancy 62.0% and limits family size 58.9%. Their source of information was mainly through health personnel in the government-owned hospitals (50.1%).
4	Ajayi, A. I., Adeniyi, O. V., & Akpan, W. (2018).	Cross sectional study	809	May- Sept 2016	Knowledge of contraception, use of contraception and reasons for non-use of contraception.	FC Awareness -93.1%, ever use-0.2% out of 645 currently using 0.2% out of 538. Reasons for non-use of FC are fear of side effects and non-availability.
5	Ijarotimi, A. O., Bakare, B., Badejoko, O. O., Fehintola, A. O., Loto, O. M., Orji, E. O., & Adegoke, A. S. (2017).	Retrospective -survey	1862	2004–2009	FC use	FC use was 0.8%.
6	Ikeako, L., Ezegwui, H., Mba, S., Iyioke, C., & Okeke, T. (2015).	Cross sectional study	313	24th November 2014 and December 15th 2014.	Knowledge of FC, use of FC,	FC awareness was 76.7% and 15.9% had used it, with most acquiring information about the device from their friends 43.8%, media 40.4%, health workers 23.3% and sex partners 7.5%. 12% used it to prevent pregnancy only, 20% used it to prevent sexually transmitted infections only while 38% used it to prevent both unwanted pregnancy and sexually transmitted infections including HIV/AIDS. About 28% used female condoms just to try it. Reasons cited for non-use are decreased satisfaction 30% and pain during sexual intercourse 30%. Others experienced difficulty in inserting female condoms into the vagina 28%, noise during sexual intercourse, 12% and failure leading to pregnancy 2%. 54% of male partners strongly approved of it, while 14% strongly disapproved.
7	Nwaokoro, J., Ede, A., Ibe Sally, N., Emerole, C., Nwuto, R., Nwaokoro, A., & Igwe, I. (2015).	Cross-sectional study	210	Not specified	Knowledge of FC, knowledge of how to use of FC, source of FC knowledge.	Awareness of FC was 61.4%, while 25.2 % said they knew how to use the FC. The students that reported that they use FC in every sexual intercourse were 3.4% it. In all, 23.6% were aware of the FC from the radio, 10.6% from television, 5.7% from books, and 6.5% from school. The largest source was from friends (53.7%). The most quoted reasons for use of FC was to protect against HIV/AIDS.
8	Ezugwu, E. C., Nkwo, P. O., Agu, P. U., Ugwu, E. O., & Asogwa, A. O. (2014).	cross-sectional study	400	March 1 to August 31, 2012	FC use and reasons for non-use.	Sixty-two participants (25.1%) used a dual-contraceptive method. None of the participants reported the use of FC. The non-use of FC observed in the present study might be due to the scarcity and in some places the non-availability, of the female condom in most health facilities in South-Eastern Nigeria.
9	Onoriode Ezire, Obi Oluigbo, Victoria Archibong, Okekearu Ifeanyi and Jennifer Anyanti (2013)	Qualitative study	78	Not specified	FC use and reasons for FC use.	FC users are 58 out of 78. Reasons for the use of FC were curiosity, to prevent unplanned pregnancy. FC provided the opportunity to meet the sexual needs of the partner irrespective of the woman's is menstrual cycle.
10	Tobin-West, C. I., Maduka, O., Onyekwere, V. N., & Tella, A. O. (2014).	cross-sectional study	900-- 427 (52.7%) females and 383 (47.3%) males;	October to November 2011	Knowledge, access, acceptability and use of FC, sources of information on FC.	Although 89.3% were aware of FC, only 8.9% had ever used one due to unavailability, high cost, and difficulty with its insertion. Nevertheless, 53.8% of the students expressed willingness to use them if offered, while 69.4% would recommend it to friends/peers. Information on FC were gotten from the media, health facilities, friends', peers and relations.
11	Asekun-Olarinmoye, E., Adebimpe, W., Bamidele, J., Odu, O., Asekun- Olarinmoye, I., & Ojofeitimi, E. (2013).	cross-sectional study	359	Not specified	Respondents' awareness and reasons for use of FC and male partners' consent.	FC awareness was 65.5% aware of FC. Among the most common cited reasons for use of FC were child spacing (63.5%) and prevention of unwanted pregnancies (59.9%). 3.6% of the male partners disapproved of it.
12	Salawu, M., & Adeyemi, A. (2013).	Retrospective -survey	5360	2007 National HIV/AIDS and Reproductive Health Survey (NARHS)	Knowledge of FC, use of FC, reasons for use and non-use, predictors of use of FC.	The women that have heard of FC were 12.6% and 5.7% knew where to get them. However, only 0.4% had ever used the female condom. Reasons for non-use were: it slipped out 0.2%, made noise 0.1% dislike 0.1%. The predictors of use of FC were age at first sexual intercourse.
13	Oladeinde, B. H., Omoregie, R., & Abdulfatai, A. (2011).	cross-sectional study	435 (261) (174)	Not specified	Knowledge and use of FC, reasons for use or not use and sources of knowledge.	Awareness of the FC was significantly higher among female undergraduate students (FUGS) (93.7%) than rural resident women (RRW) (5.2%. No significant difference was observed in level of use of the FC between FUGS (1.9%) and RRWM (0%). Reasons for non-use are unavailable 5.7%, ashamed to buy product 4.8%, belief forbids its use 1. %, partners disapproves 4.7%. While the reasons for use are to prevent pregnancy 88.6% in FUGS and 55.5% in RRW, tO Prevent HIV transmission 100% in FUGS and 33.3% in RRW, to prevents STIs 75.1% in FUGS and 11.1% in RRW. Sources of knowledge includes Internet 42.0%, media 86.9% and friends 15.1%.
14	Olugbenga-Bello, A. I., Adekanle, D. A., Ojofeitimi, E. Ï., & Adeomi, A. A. (2010).	cross-sectional study	201	Not specified	Knowledge, use, reasons for use and no use of barrier contraception (B C)	Knowledge of FC 87.4% used the FC 0.6%. Reasons for use are to prevent STI and unwanted pregnancy. Reasons for not were religion, uncertainty about safety, culture, decreased sexual pleasure, and belief.
15	Abalaka G. (2008)	cross-sectional survey	392	Not specified	Awareness, knowledge, usage and reasons for use and non-use of FC. Sources of information on FC.	Less than half 40.6% and 27.8% had ever heard of and seen female condom respectively. Only 4.3% of those had ever used it. Seventyone percent of those who had ever used female condom intended to continue, while 29.4% were unsure. Reasons for use are to prevent unwanted pregnancy (80%) and prevention of STls (28.4%). While reason for non-uses includes the fear of FC dropping into woman's body leading to death (29.7%), its oily nature (23.9%), the balloon-like look (23.9%) and inhibiting natural sexual feeling (14.2%). Information on FC were from health facilities (41.3%), media at 32.3%, 24.5% respondents heard about FC from their neighbours, while 15.5% from academic institution.
16	kunlola, M., Morhason-Bello, I., Owonikoko, K., & Adekunle, A. (2006).	cross-sectional survey	850	Not specified	Knowledge and use the FC, factors affecting the use, sources of information on FC and male partner's consent.	Over 80% had knowledge of the FC as a form of contraception and the majority of them learnt about it through the mass media (39.9%) and health workers (34.4%). However, only 11.3% had ever used the female condom. The majority (42.7%) of the sexual partners of female condom users approved of it; however, 39.6% of sexual partners disapproved while 17.7% were indifferent. Most (40%) using it to prevent both unwanted pregnancy and sexually transmitted infections including HIV (STI/HIV); 27.1% to prevent pregnancy alone and 19.8% to prevent STI including HIV only, while 12.5% used it on a trial basis. Reasons for not use: lack of sexual satisfaction (30.2%). Other problems encountered included difficulty in inserting it into the vagina (21.7%), pain during sexual intercourse (5.2%) and method failure resulting in pregnancy in two (2.8%) of the respondents
17	Adeyemi, A. S., & Adekanle, D. A. (2009).	retrospective review	1355	January 2001 and December 2006	FC use, reasons for use and sources of information on FC.	FC use was 0.2%. Reasons for use of FC 37.8% to prevent further pregnancy, 56.9% for child spacing and 5.6% not sure, if they want kids again. Sources of information on FC were family planning clinic personnel 76.7%, media (print ad electronic) 11.2%, friends and relatives 9.4% and community health workers 2.7%.

Abbreviations: FC = female condom

**Table 1b:** Summary of the outcomes assessed across the studies

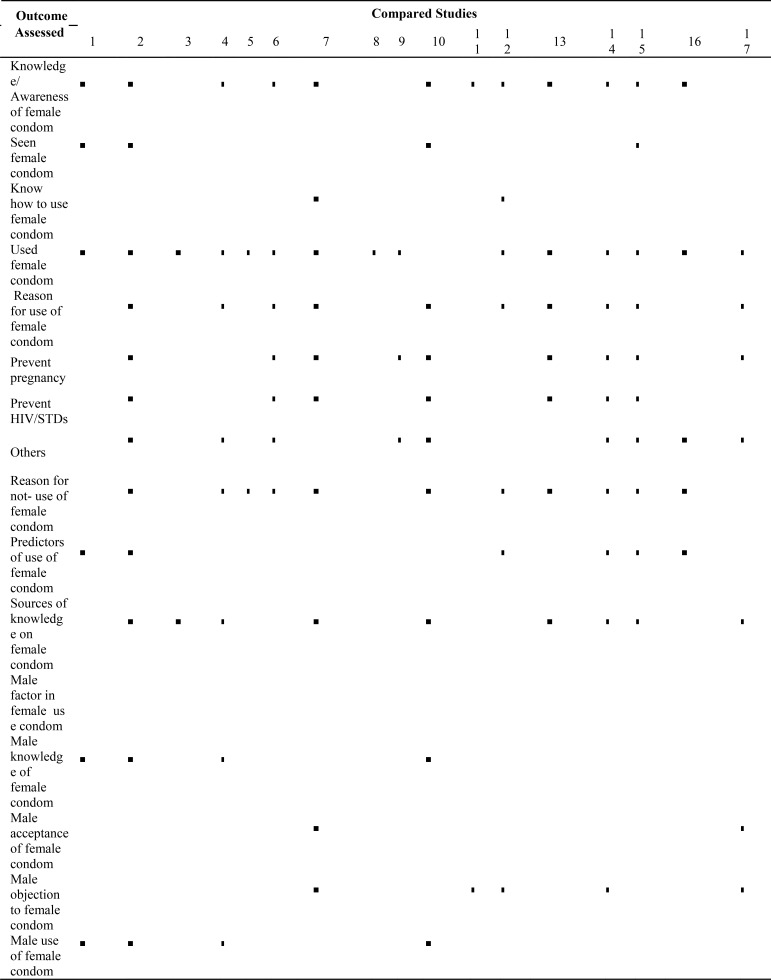

### Particip ants and Statistical Analysis

The number of participants in each study ranged from 78 to 5,360 women. The seventeen studies included 14,949 women and 1,225 men. Graphpad Prism statistics software, version 7 (California, USA) was used to analyse the data. Descriptive statistics for continuous data is presented by mean ± standard deviation. To determine the statistical difference between study groups and across study groups, the one-way ANOVAest was used. A p-value of < 0.05 was considered statistically significant.

### Female condom awareness and use

**Fig. 2:**
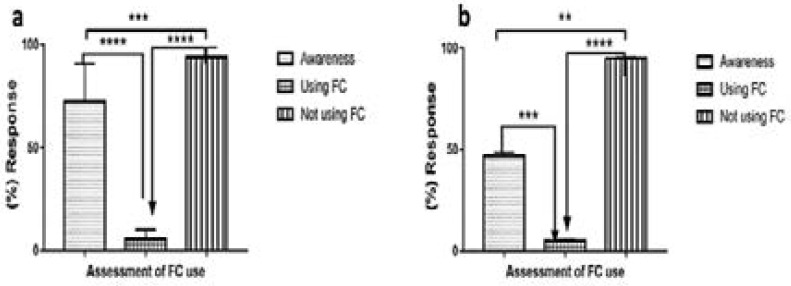
Female condom awareness and use in (a) female and (b) male respondents.

Figures 2a & 2b present the assessments on FC use in males and females respectively. One-way ANOVA analyses showed significant differences between females [F (2, 27) = 69.34, p < 0.0001] and males [F (2, 6) = 84.21, p < 0.0001]. Significant differences were also noted in the knowledge versus FC use for females (p < 0.0001) and males (p < 0.001); knowledge versus FC not used showed significant differences in both females (p < 0.0001) and in males (p < 0.01). In addition, a significant difference (p < 0.0001) was observed between FC use versus FC not use in both genders.

Twelve studies reported awareness or knowledge of FC, with percentage awareness ranging from 93.7% amongst female undergraduate students 19 to 40.6% amongst married women 20. In this review, after meta-analysis, we reported an average awareness of 72.5% and 46.9% amongst the female and male respondents, respectively (Fig. 2). Seven of the studies were conducted amongst undergraduate students; three of which were in the South-Western region, an average awareness of 87.03% (range: 80–93.7%) 19,21,22. The South-South region followed with an average awareness of 89.3% conducted among 900 respondents made up of both males and females 14. The South-Eastern region reported lowest awareness rates in the three sets of studies; 76.7%, 62.3%, and 61.4% 23–26, with an average of 66.8%. The three studies that reported awareness among women showed a wide variation of awareness: ranging from 40.6% to 93.1% in married women^20^,^27^ and 65.5% among women of unspecified marital status 28. The variation could be due to the periods of the studies; since the lowest awareness was reported in 2008 and highest awareness in 2016. However, a recent study conducted in 2019, reported low awareness of 47.9%, among street youths in the South-Western region 17. The two studies that only interviewed males also reported low awareness rates of 48.15 and 45.6%^14^,^17^.

Despite the high levels of awareness among the female respondents, the use of the FC was low and ranged between 0.2% and 15.9%, with an average of 5.5% (Fig. 2a). Males also reported low levels of partner use, and an average of 5.05% (Fig. 2b). Use was similar in both studies that interviewed male respondents, which reported use of 5.5% and 4.6%, respectively 14,17. Of the fifteen studies that reported the use of the FC, the 2007 National HIV/AIDS and Reproductive Health Survey (NARHS) reported the lowest use of the FC (0.4%) 2 9. Seven studies, which were conducted among undergraduate students showed a wide range of frequency of FC use between 1.9% and 11.3% in the South-Western region 19,21,22. A similar pattern was reported from the South-Eastern region where use ranged from 3.0% to 15.9% 23,25,26. The trend is not different in the South-South region, with an 8.9% average FC use 14. The percentage is lower among married women (4.3% & 0.2%), street youth (4.3%) and 0.8% among men and women of reproductive age 17,20,27,30.

### Reasons for the use and non-use female condom Reasons for the use of the female condom.

**Fig. 3:**
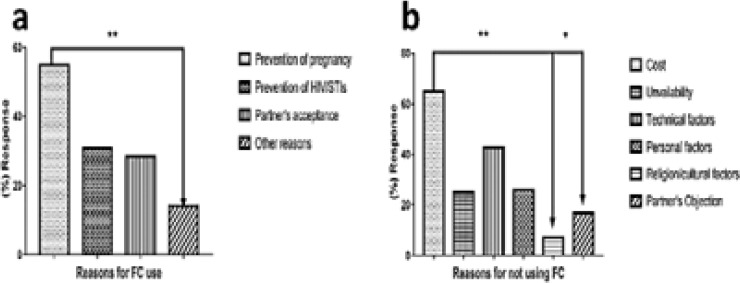
Reasons for the use and non-use of the female condom.

Figure 3a presents the reasons for FC use by the female respondents., which are categorised into: prevention of pregnancy, prevention of HIV/STIs, partner's acceptance and any other reason/s. One-way ANOVA showed significant differences between the various reasons for FC use among female respondents [F (3, 8) = 7.906, p = 0.009]. A significant difference was also noted for using the FC for the prevention of pregnancy versus other reasons for not using the FC (p < 0.006). The main reasons for using FC varied among the respondents. While most male respondents cited the prevention of STIs, including HIV as a reason for using the FC 26, others cited unwanted pregnancy 21. Slightly over half of the female respondents (55%) 14,20,22,31,32 used it because they wanted to prevent unwanted pregnancies (Fig. 3a), 31% used it to prevent STIs, including HIV 21,23,26 and 28.3% used it based on their male partner's acceptance 14,21,25 (Fig. 3a). Some (14%) used it for other reasons (Fig. 3); such as having sex with a non-regular partner (22.1%) 25 child spacing (56.9%) 32, to meet the sexual needs of the partner irrespective of the woman's menstrual cycle 33 or for fun (17.7% and 28%, respectively) 22,23. Another interesting reason for using female condoms among female students was that this offered independence as well as a sense of self-empowerment (19%) 14.

### Reasons for not using female condoms

Figure 3b presents the reasons for not using the FC by female respondents. One-way ANOVA analysis showed significant differences [F (5, 13) = 5.195, p = 0.0077] between the different reasons provided for not using FC by the female respondents. Significant differences were noted between the costs versus religious/cultural factors (p < 0.007) and between costs versus personal factors (p < 0.02). The reasons for non-use of FC are discussed under the following themes:

Cost and availability: Costs constituted 65% of the reasons for non-use of FC (Fig. 3b) 14, while the unavailability of FC made up about 25.3%. 14,21,25.

Technical factors: These included not knowing how to use the FC 25, difficulty in inserting the FC into the vagina, noise during sexual intercourse, its oily nature, the balloon-like looks and failure leading to pregnancy 23. Personal factors: The fear of side effects was the most reported reason for non-use; some women believe that contraceptives are harmful to the body 27, cause decreased sexual satisfaction 19, pain during sexual intercourse and fear 19,21,23. Many had a low perception of HIV or STI risk and concerns about the length and aesthetic appearance of the condoms were also raised 14. Female students either did not use it because of male partner objection or preference for other methods of contraception 19,25.

Religious/cultural factors: One reason for non-use of FC was religious beliefs; some respondents claimed that their religion bans the use of the FC. While others did not use it for cultural reasons, the use of FC was normally seen as taboo in some cultures^19^,^21^,^25^,^26^.

### Sources of knowledge and awareness of the female condom

**Fig. 4:**
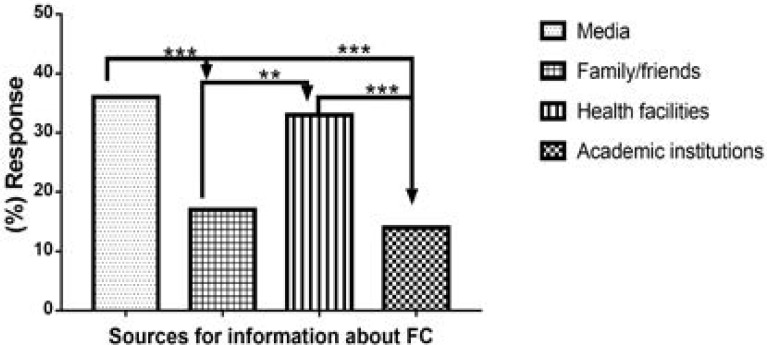
Sources of information on the knowledge and use of the female condom.

Figure 4 presents the sources of information on FC knowledge. One-way ANOVA analyses showed significant differences [F (3, 8) = 32.89, p < 0.0001] between the sources of information on FC knowledge. Specific significant differences were also noted between obtaining information from the media versus family and/or friends (p < 0.001), media versus academic institutions (p < 0.001); health facilities versus family and/or friends (p < 0.01); health facilities versus academic institutions (p < 0.001). In Nigeria, the discussion of sex is considered the exclusive preserve of adults 34. This practice might be responsible for the family contributing a very low percentage, sometimes as low as 0%, as a source of knowledge about contraception and the FC in particular 21. The media assumes the primary source of information on contraception at 36% 19,25–27,32,35, followed closely by health facilities at 33% 22,31,32, family and/or friends at 17% 17,23 and from academic institutions at 14%.

## Discussion

In this review, we assessed the awareness and use of the FC among Nigerian men and women. Available evidence suggests discordance between awareness and the use of female condoms. Despite high levels of awareness, especially among the female respondents, the use of the FC has remained extremely low, even among the young educated female undergraduate students. The high level of awareness about the FC did not translate to a positive attitude towards its use. A similar study among South African women reported low use among more educated females, which in their case was counteracted by their higher use of the male condom, due to better negotiation skills with the latter 36. Our results support the findings that correct knowledge of health issues may not always translate into appropriate preventive health actions.

Nevertheless, this review highlighted significant challenges related to FC availability, affordability, ease of insertion, noise during sexual intercourse, its oily nature, the balloon-like looks and failure leading to pregnancy. Other challenges mentioned included the fear of side effects, decreased sexual satisfaction, pain during insertion and during sexual intercourse, the length and aesthetic appearance of the condoms, partner disapproval, preference for other methods of contraception, religious and cultural believes. These could be responsible for its unpopularity among university students. Some studies have also documented similar challenges, in developed countries 14,36–38. Despite these challenges, however, there were indications of considerable prospects for acceptability and use of female condoms among the students interviewed, as over two-thirds expressed optimism and willingness to try out the condoms if offered as a trial or made available and will even introduce it to their friends and peers 14.

The primary reasons given by the respondents for their use of the FC are fear of pregnancy (55%), STIs (31%) and other reasons (14%). Female condoms are significant in the quest for a sustainable approach in the prevention of HIV and other STIs and unintended pregnancies 8. Studies have shown that condoms provide an impermeable barrier to particles the size of sperm and STI pathogens, including HIV 39,40. The use of condoms, consistently and correctly, is highly effective in preventing the sexual transmission of HIV. To ensure safe and effective use, FCs must be produced according to specifications, international standards, and quality assurance procedures established by WHO^41^. Adequate investment in and further scale-up of condom promotion is required to sustain responses to HIV, other STIs, and unintended pregnancy. There is a need for a robust and consistent advocacy to make the FC available either free or at an affordable cost. Moreover, there is a need to intensify awareness to erase erroneous perceptions of the FC, using the media and health facilities, which constituted 36% and 33% of the significant sources of information, respectively. Notably, in recent times, social media has played a leading role as an avenue for disseminating reproductive health information amongst youth. Interpersonal communication activities, such as rallies, roadshows, one-on-one educational contacts, group meetings in strategic places like markets, religious houses, schools, and seminars should be encouraged. However, the FC is not an all-or-nothing option, but it should augment the protective options available, there by increasing the choices available to wome^36^.

The limitations of this review include paucity of data on FC from the Northern regions of the country and general lack of data on male's perspectives of FC. This can be overcome through a comprehensive national study on FC to include both genders.

## Conclusion

We conclude that despite the high levels of awareness, especially among the female respondents, the use of the FC has remained extremely low. Therefore, there is the need for deliberate and vigorous advocacy to increase FC demand and increase its availability and affordability. There is also a need to erase the erroneous perceptions associated with the female condom.

## Data Availability

The datasets analysed during the current study are publicly available and were obtained by searching Google Scholar and PubMed.
